# Spatial scale of non-target effects of cotton insecticides

**DOI:** 10.1371/journal.pone.0272831

**Published:** 2023-05-10

**Authors:** Isadora Bordini, Steven E. Naranjo, Alfred Fournier, Peter C. Ellsworth

**Affiliations:** 1 Department of Entomology, Maricopa Agricultural Center, University of Arizona, Maricopa, AZ, United States of America; 2 USDA-ARS, Arid-Land Agricultural Research Center, Maricopa, AZ, United States of America; University of Saskatchewan College of Agriculture and Bioresources, CANADA

## Abstract

Plot size is of practical importance in any integrated pest management (IPM) study that has a field component. Such studies need to be conducted at a scale relevant to species dynamics because their abundance and distribution in plots might vary according to plot size. An adequate plot size is especially important for researchers, technology providers and regulatory agencies in understanding effects of various insect control technologies on non-target arthropods. Plots that are too small might fail to detect potential harmful effects of these technologies due to arthropod movement and redistribution among plots, or from untreated areas and outside sources. The Arizona cotton system is heavily dependent on technologies for arthropod control, thus we conducted a 2-year replicated field experiment to estimate the optimal plot size for non-target arthropod studies in our system. Experimental treatments consisted of three square plot sizes and three insecticides in a full factorial. We established three plot sizes that measured 144 m^2^, 324 m^2^ and 576 m^2^. For insecticide treatments, we established an untreated check, a positive control insecticide with known negative effects on the arthropod community and a selective insecticide. We investigated how plot size impacts the estimation of treatment effects relative to community structure (27 taxa), community diversity, individual abundance, effect sizes, biological control function of arthropod taxa with a wide range of mobility, including *Collops* spp., *Orius tristicolor*, *Geocoris* spp., *Misumenops celer*, *Drapetis* nr. *divergens* and *Chrysoperla carnea s*.*l*.. Square 144 m^2^ plots supported similar results for all parameters compared with larger plots, and are thus sufficiently large to measure insecticidal effects on non-target arthropods in cotton.

Our results are applicable to cotton systems with related pests, predators or other fauna with similar dispersal characteristics. Moreover, these results also might be generalizable to other crop systems with similar fauna.

## Introduction

Plot size is of practical importance for anyone designing experiments involving mobile species, regardless of the study purpose. Because natural arthropod communities range freely over large agricultural areas, observations from small areas could be quite different from the dynamics and patterns that occur in large areas. Thus, plots need to be as large as practically possible for experiments to be applicable to commercial scales [1–3].

Choosing an adequate plot size is particularly important in non-target arthropod assessments involving insect control technologies, e.g. insecticides and transgenic *Bt* crops. Several authors have demonstrated that the distribution and abundance of arthropods in plots varies according to plot size [1–6]. This variation can be especially problematic if plots are too small to detect harmful effects of these technologies on non-target arthropods due to their movement and redistribution among plots, or from untreated areas and outside sources [2].

However, larger plots necessarily result in higher costs associated with land rental, water, labor and equipment, and might not be tenable when there is a limited amount of seed in the case of regulated trials. Plots need to be as large as practically possible while balancing the goals of accurately measuring gross effects on non-target arthropods and using on an area suitable for the ecological attributes of the arthropod community, especially mobility [2, 7].

Some authors have suggested that few studies have been conducted in field plots of sufficient size to understand potential disruptions of insecticidal technologies on biological control agents and other non-target organisms [3, 8–10]. Such studies need to be conducted at a scale that is relevant to species dynamics in any integrated pest management (IPM) study that has a field component, and to allow regulatory bodies to properly evaluate data submitted for insecticide or trait registration by technology providers. While harmful effects of transgenic crops on non-target arthropods are probably relatively small compared with insecticides [11], even minor issues with experimental design could compromise the ability to detect non-target effects [2, 12].

We conducted a 2-year field experiment to estimate the optimal plot size for non-target arthropod studies in the Arizona cotton system. Our IPM program is heavily dependent on selective insecticides and the conserved biological control provided by key predators of our two key pests, *Lygus hesperus* Knight and whiteflies, *Bemisia argentifolii* Bellows and Perring *(= B*. *tabaci* MEAM1) [13–17]. We investigated how plot size impacted our ability to measure treatment differences using multiple metrics including individual predator species abundance, arthropod community structure and diversity, and biological control function.

## Materials and methods

### Experimental design

Experiments were conducted at the University of Arizona’s Maricopa Agricultural Center, Maricopa, AZ, United States, in 2017 and 2018. Cotton, *Gossypium hirsutum* L., was planted on 1 June 2017 and 4 May 2018, and grown in accordance with agronomic practices for the area. The variety planted in both years, DP1549B2XF, was a Bollgard II® XtendFlex® variety (Bayer Company, MO, USA), which provides resistance to lepidopteran insects and tolerance to dicamba, glyphosate and glufosinate herbicides.

A randomized complete block design was used in both years. Plots were established in a single field site about 3 ha in size, subdivided into four blocks. Within blocks, treatments were randomly assigned to plots.

Experimental treatments consisted of plot sizes and insecticides in a full factorial. Three square plot sizes were established: a “small” plot: 12 m long by 12 m (12 rows; 144 m^2^); “medium” plot: 18 m long by 18 m (18 rows; 324 m^2^); “large” plot: 24 m long by 24 m (24 rows; 576 m^2^) with ca. 1 m row spacing, and 3 m unplanted alleys. Two controls were established, a negative control, an untreated check (water only, UTC), and a positive control (a broad-spectrum insecticide) with known negative effects on the arthropod community [17] This positive control enabled us to assess the ability of our experimental design to detect an expected effect on the arthropod community, and was implemented as acephate (Orthene® 97P, Amvac Chemical Corporation, California, USA) at 1120 g a.i./ha. Acephate has commercial activity against *L*. *hesperus*, but essentially no effect on *B*. *argentifolii*. A third treatment consisted of a selective insecticide that targets *B*. *argentifolii* and with proven non-effects on the arthropod community [17]. The selective insecticide was flupyradifurone (Sivanto™ 200SL, Bayer Crop Science, North Carolina, USA) applied at 202 g a.i./ha.

The trial was sprayed with a six row (6 m) tractor-mounted boom sprayer (TJ69-8003VS TeeJet® spray tips, two over the top nozzles per row) at a volume of 112.5 l/ha. To avoid drift, insecticides were sprayed directly to plots during calm weather conditions, using low spray boom heights and reduced sprayer ground speed.

Calendar sprays with acephate and flupyradifurone treatments were made every 14 days for a total of 3 sprays at their highest labeled rates during the flowering period. These were scheduled sprays for the purposes of non-target evaluations, and therefore they were not based on needs dictated by action thresholds for pest control. Spray dates were 1 August, 15 August and 29 August in 2017, and 2 August, 16 August and 30 August in 2018. A plant growth regulator (mepiquat pentaborate, Pentia™ 99 g ai / l, BASF, Texas, USA) was sprayed in 2017 according to cotton commercial guidelines to manage the balance between vegetative and reproductive growth for cotton production. These plant growth regulator sprays were not necessary in 2018.

### “Maintenance sprays” for prey uniformity

Ideally, pest levels should be comparable among insecticidal treatments as pests can change plot conditions (i.e., flower loss due to Lygus damage or excess honeydew due to whiteflies) that might affect arthropod dynamics, thereby causing gross changes across treatments that could potentially mask the effects of the intended treatments on arthropods [17]. Bordini et al. [17] deployed “maintenance sprays” when testing the selectivity of insecticides that controlled Lygus or whiteflies. The objective of these “maintenance sprays” was to preserve prey parity (*L*. *hesperus* and *B*. *argentifolii*) as much as possible among insecticidal treatments by spraying other insecticides that selectively targeted either whiteflies or Lygus [17]. Since insecticidal treatments provide control of different pests and one treatment is broad-spectrum (acephate controls *L*. *hesperus*) and the other one is selective (flupyradifurone controls *B*. *argentifolii*), we anticipated that there would be disparities among major arthropod prey. Thus, we tried to maintain comparable levels of *B*. *argentifolii* and *L*. *hesperus* in all plots as much as possible with these “maintenance sprays”. The most convenient metric for those levels is the action threshold for each pest. Thus, these sprays targeted *L*. *hesperus* and *B*. *argentifolii*, and were deployed at economic threshold levels based on standard sampling methods for these pests [18, 19].

Maintenance sprays for *L*. *hesperus* control were deployed in the untreated check and flupyradifurone treatments, but not in the positive control as acephate has commercial activity against Lygus (S1 Table in S1 File). The maintenance sprays for *L*. *hesperus* control were a selective insecticide (flonicamid, Carbine® 50WG, 98 g ai/ha, FMC Corporation, Pennsylvania, USA) applied twice in 2017 (10 and 24 August) and three times (2, 16, 30 August) in 2018. Maintenance sprays for *B*. *argentifolii* control were deployed once in the positive control and once in the untreated check in both years. We sprayed the selective insecticides, pyriproxyfen (Knack® 0.86EC, 75 g ai/ha, Valent, California, USA) on 24 August 2017, and buprofezin (Courier® 3.6SC, 390 g ai/ha, Nichino America, Delaware, USA) on 30 August 2018. As flupyradifurone has whitefly activity, no maintenance sprays were required against whiteflies; density levels there never exceeded the threshold.

### Arthropod sampling

Lygus (nymphs and adults) and other arthropods were sampled concurrently with a standard 38 cm diameter sweep net. Sampling was done at three, seven and 13 days after each spray (a total of 9 weekly dates over the season each year). Twenty-five sweeps per plot were used in the small plot, 2 sets of 25 sweeps (50 sweeps) in medium plots, and 4 sets of 25 sweeps (100 sweeps) in large plots. This intensity of sampling was similar across different plot sizes to ensure similar removal of arthropods. All data were standardized to 100 sweeps, because this is the unit of measurement used for *L*. *hesperus* and predator sampling in our system [16, 18].

Densities of *B*. *argentifolii* were sampled at three and seven days after each spray, for a total of six samples each year. Ten leaves from the fifth mainstem node below the terminal per plot were randomly selected to estimate adult density and collected to estimate egg and nymph density in the laboratory. Adult density was estimated by counting individuals on the underside of leaves *in situ* [19]. Nymph and egg densities were estimated by counting individuals in the laboratory under magnification on a 3.88 cm^2^ disk taken from these leaves [20, 21].

Densities of 27 additional arthropod taxa were estimated, including key arthropod predators in our system (Vandervoet et al., 2018); *Collops quadrimaculatus* (Fabricius), *C*. *vittatus* (Say), *Orius tristicolor* (White), *Geocoris punctipes* (Say), *G*. *pallens* Stål, *Misumenops celer* (Hentz), *Drapetis* nr. *divergens* Loew and *Chrysoperla carnea s*.*l*. (Stephens). Samples were frozen and later counted in the laboratory using a dissecting microscope. These are all generalist predators. *Collops* spp. are soft-winged flower beetles from the family Melyridae. The hemipterans *Geocoris* spp. (family: Geocoridae) and *Orius* spp. (family: Anthocoridae) are piercing-sucking predators. *M*. *celer* are commonly known as “crab spiders” and belong to the family Thomisidae. *C*. *carnea* is a species of green lacewing from the Chrysopidae family.

### Biological control function

#### Predator to prey ratios

The key predators mentioned above were used to calculate predator to prey ratios as the quotient of each species per 100 sweeps to the number of *B*. *argentifolii* adults per leaf or large nymphs per leaf disc. We estimated eight predator to prey ratios as follow: *M*. *celer*/*B*. *argentifolii* adults, *M*. *celer*/*B*. *argentifolii* large nymphs, *D*. nr *divergens*/*B*. *argentifolii* adults, *D*. nr *divergens*/*B*. *argentifolii* large nymphs, *O*. *tristicolor*/*B*. *argentifolii* adults, *C*. *carnea* larvae/*B*. *argentifolii* adults, *Collops* spp./*B*. *argentifolii* large nymphs and *G*. *punctipes*/*B*. *argentifolii* large nymphs. For *Geocoris spp*., evaluations were done only with *G*. *punctipes* because *G*. *pallens* Stål densities were very low throughout the years of these trials. We chose these ratios because at certain levels they independently indicate functioning whitefly biological control in our system [16, 17, 22, 23]. We also calculated the proportion of time that these ratios were at or above functioning biological control over the season [17].

#### Sentinel prey

A novel *in situ* sentinel prey method was developed to measure biological control function along with other sources of in-field mortality for *B*. *argentifolii* eggs and fourth instar nymphs based on procedures developed for life tables of sessile insects [24, 25]. Whiteflies are convenient and realistic as sentinel prey, because nymphs and eggs are immobile on leaves, abundant in the field, and natural enemies readily feed on them. They also are the most important pest subjected to biological control in our system. We used fourth instar nymphs because they 1) are easily seen in the field, 2) are the last instars prior to adult emergence and thus it is possible to distinguish successful emergence of adults from marks of mortality, 3) mortality is greatest during the fourth stadium, followed by mortality during the egg stage [24], 4) insecticide thresholds in our system are based on large nymph numbers in addition to adults, and 5) the majority of our primary natural enemies feed on nymphs. We used eggs because this stage is subject to the second highest mortality level, and some of our key predators (*O*. *tristicolor*; *Geocoris spp*. and *C*. *carnea* larvae*)* feed on whitefly eggs [24].

Three cohorts of at least 25 newly-laid live eggs (< 1 day old) per plot were established on 1, 16 and 29 August in 2018. The first two cohorts were established the same day of the sprays, and the third cohort was established the day before the last spray. Two cohorts of at least 25 newly molted fourth instar live nymphs, appearing flat and translucent, per plot were established on 2 August in three replicate plots, and 29 August in all plots in 2018. The first cohort was established the same day of the first spray, and the second cohort was established the day before the last spray.

Leaves with nymphs were collected 4–5 days and leaves with eggs at 7 days after establishment and taken to the laboratory for inspection. These intervals correspond with developmental times for these stages in a typical Arizona summer. Eggs and nymphs were examined to determine in-field mortality sources using a dissecting scope in the laboratory. A single observer made all determinations within a single block. Mortality was recorded as due to predation, parasitism, inviability or eggs that failed to hatch, dislodgement and unknown. Dislodgement can be due to weather like rain and/or wind or to chewing predation by beetles mainly from *Collops* spp. or Coccinelidae. This type of predation usually removes all trace of the individual. However, in rare cases we could observe partial nymphal cadavers or egg pedicels still anchored on leaves [24]. Predation was mainly due to the sucking predators *Geocoris* spp., *Orius* spp. and *C*. *carnea* larvae as shown in previous studies [24]. These predators evacuate the content of nymphs and eggs, leaving a transparent nymph cuticle or egg chorion on the leaf.

Different factors that affect *B*. *argentifolii* mortality occur simultaneously and so there is no obvious temporal sequence of mortality. Thus, mortality from one factor can conceal the action of a previous factor [24]. To account for this and estimate mortality accurately, marginal mortality rates were calculated for each factor ([25–28]; see [24] for formulas).

### Diversity indices

Arthropods (43 taxa) collected from sweep samples were used to calculate Species Richness (S), the Shannon-Wiener Diversity Index (H), the Effective Number of Species associated with the Shannon Diversity Index (ENS) and the Shannon Evenness Index (J). These metrics were calculated for every treatment plot.

### Statistical analyses

We used a mixed-model, repeated measures analysis of variance (JMP® Pro 14.2, SAS Institute Inc., Cary, NC) to test for treatment differences (insecticide and plot size) affecting the abundance of the six key predators and key pests over the season in both years. We also used this model to test for treatment differences in diversity metrics. The model included fixed effects of plot size, insecticide, year and sampling date (repeated measure) and their interactions. Block and associated interaction terms were considered random effects. The covariance structure used was AR(1). We used the proportion of maximum scaling method (POMS), which transforms each scale (predators’ scale in each year) to a common metric running from 0 (= minimum possible) to 1 (= maximum possible) [29], to minimize expected year effects in abundance of the predatory arthropods and to enable more consistent comparisons between years. We compared the mean weekly abundance of the six key predators, pests, diversity metrics using Dunnett’s test within each year. The untreated check for large plot size was the standard.

Analyses were done for all sample dates after the first application of insecticides. Before insecticidal application, pre-counts of arthropod densities were not statistically different. To facilitate the visualization of patterns, we graphically represented pest and predator abundance using cumulative arthropod-days over the season using the trapezoidal rule [30].

We calculated the proportion of dates that each key predator to prey ratio was above functioning biological control levels in our system [17]. These data were analyzed using a mixed-model that included fixed effects of insecticide treatment and year; the block variable and associated interaction terms were entered as random effects (JMP® Pro 14.2, SAS Institute Inc., Cary, NC). The same model was used to test for treatment differences (insecticide and plot size) in marginal mortality for nymphs and eggs. In all cases, Dunnett’s test was used to compare results from treatments with the large plot untreated check.

We examined the main effects of plot size on the entire arthropod predator community through Principal Response Curves (PRC), a time-dependent, multivariate analysis that depicts arthropod community trends over time for each treatment relative to a control [31–34]. We examined the main plot size effects using the large plot size as the standard. PRCs use a distribution-free *F* type test based on sample permutation to test for statistical significance in patterns. Principal response analyses were done in CANOCO v5 (Microcomputer Power, Ithaca, NY, USA).

Finally, we estimated Hedge’s d effect sizes between means from arthropod abundance (key predators, *Lygus* spp. and *B*. *argentifolii*) in each insecticidal treatment (positive control and flupyradifurone) relative to means in the untreated check for each plot size.

## Results

### Individual predator abundance

**Fig 1:**
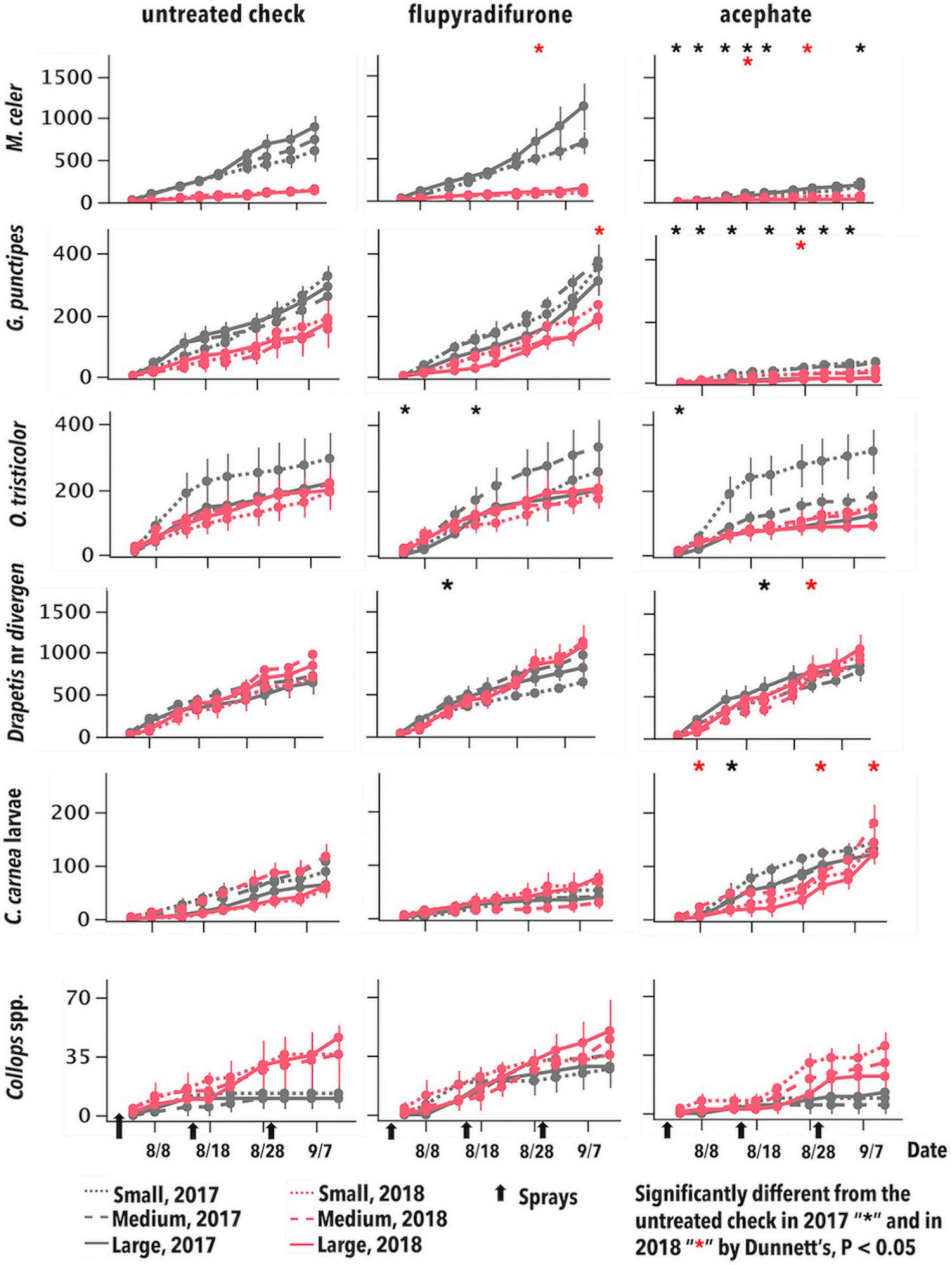
Post-treatment, cumulative mean insect-days (error bars = S.E) for arthropod predators per 100 sweeps during two growing seasons in Maricopa, AZ. Asterisks correspond to treatment means for the main effect of insecticides that were significantly different from the untreated check by Dunnett’s, P < 0.05, by date and year; plot size and its interactions were not significant.

Plot size and its interactions were not significant for key predator abundance (P > 0.05; Table 1; Fig 1). Predator abundance in the flupyradifurone treatment was either statistically greater or not different from the UTC most of the time, being lower than the UTC in only two instances (once for *O*. *tristicolor* after the first spray in 2017, and once for *M*. *celer* after the third spray in 2018). The negative control, acephate, supported much lower predator densities as expected. The results from the negative control provide strong evidence that the experimental design was robust enough to measure the known destructive effect of this insecticide.

**Table 1:** Fixed effect F-values for mean arthropod predator abundance (per 100 sweeps) over two years. See Fig 1 for plots of density over the season.

Fixed Factors	DF	*M*. *celer*	*Geocoris punctipes*	*Orius tristicolor*	*Drapetis nr divergens*	*C*. *carnea larvae*	*Collops spp*.
Insecticide	2, 53.9	26.69***	41.17***	2.70	3.13	28.20***	4.43*
Plot Size	2, 53.9	0.83	1.09	1.84	0.54	2.07	0.03
Year	1, 53.9	5.55*	7.04*	3.74	38.53***	0.14	13.14***
Date	8, 383.7	4.59***	7.36***	18.55***	27.43***	5.70***	4.29***
Insecticide*Plot Size	4, 53.9	1.66	0.32	0.49	0.59	2.23	0.43
Insecticide*Date	16, 410.1	1.52	3.17***	1.49	0.78	3.30***	0.83
Insecticide*Year	2, 53.9	0.71	0.89	0.06	0.69	0.87	1.51
Insecticide*Year*Date	16, 410.1	1.78*	1.16	2.01*	1.12	2.80**	0.56
Plot Size*Year	2, 53.9	1.15	0.06	0.76	0.04	0.55	0.03
Plot Size*Date	16, 410.1	0.68	0.69	1.34	1.10	0.91	0.97
Plot Size*Year*Date	16, 410.1	0.85	0.48	1.54	1.25	0.67	0.63
Date*Year	8, 383.7	2.71**	2.46*	12.72***	29.69***	6.64***	5.98***
Insecticide*Plot Size*Date	32, 421.8	1.00	0.84	1.24	0.56	0.94	1.07
Insecticide*Plot Size*Year	4, 53.9	0.29	0.02	0.66	0.39	0.94	0.67

Repeated-measures ANOVA

* *P* < 0.05

** *P* < 0.01

*** *P* < 0.001. DF are approximated.

### Non-target arthropod community dynamics

**Fig 2:**
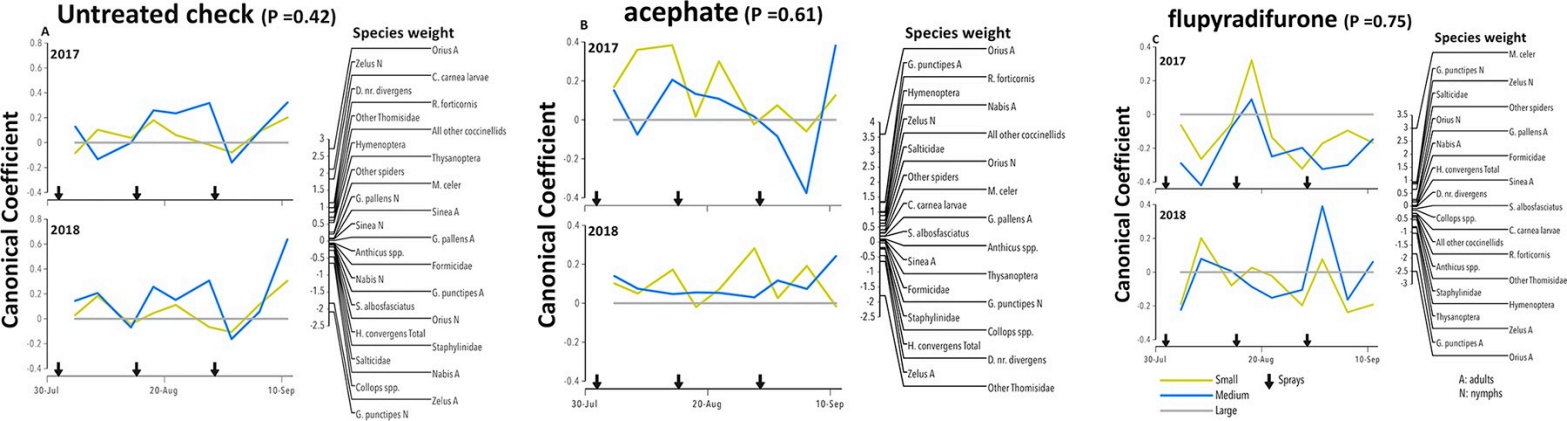
Principal response curves (PRC) showing plot size effects on the arthropod community for the untreated check (A), the positive control acephate (B) and flupyradifurone (C) relative to the large plot size (y = 0 line) during two growing seasons in Maricopa, AZ. The P-values denotes the significance of the PRC analysis in comparison with the large plot size over all dates based on an F-type permutation test. The product of the species weight and the canonical coefficient for a given plot size and time estimates the natural log change in density of that species relative to the large plot size. The greater the species weight the more the response for that species resembles the PRC. Negative weights indicate an opposite pattern, and weights between −0.5 and 0.5 indicate a weak response or a response unrelated to the PRC.

The Principal Response Curve (PRC) depicts the effect of small and medium plot sizes relative to the large plot size for the untreated check, positive control and flupyradifurone separately (Fig 2). PRCs based on the first axis of redundancy analyses were not significant (P > 0.05) in any comparison (Fig 2). We also examined each insecticide compared to the untreated check for each plot size to further understand community effects (S1-S4 Figs in S1 File). As expected, the arthropod community was significantly reduced in the negative control relative to the UTC in both years and in all plot sizes (P < 0.05) (S1-S3 Figs in S1 File). Again, this validates our experimental design because we were able to clearly measure the known destructive impacts of the negative control, acephate, on the arthropod community in all plot sizes.

### Whitefly and Lygus target pest abundance

Plot size and its interactions were not significant for *B*. *argentifolii* adults and large nymphs (P > 0.05) (S2 Table in S1 File). The main effect of plot size was not significant for *B*. *argentifolii* small nymphs and eggs. However, the interaction plot size*year*date was significant for small nymphs, and the same interaction along with insecticide*plot size*date were significant for eggs (P < 0.05). These are not ecologically relevant interactions as temporal effects were expected due to yearly and weekly changes in abundance, and the second interaction was due to only one contrast over twelve dates (S5 Fig in S1 File).

Plot size and its interactions were not significant for *L*. *hesperus* nymph abundance (P > 0.05) (S3 Table in S1 File). The main effect of plot size was significant for Lygus adult abundance only (P < 0.05), where adult abundance was significantly lower in the small plot compared with the large plot (P < 0.05); however, the maximum differences in abundance between plot sizes were 2–5 adults per 100 sweeps. Adults ranged from about 4 to 72 adults per 100 sweeps over the course of 18 sampling dates in this two-year study. Given this and the fact that we were able to detect insecticide effects on Lygus in small plot sizes (insecticide*plot size was not significant), the statistically significant difference identified between adult abundance in large compared to small plots is not likely to be ecologically important.

**Fig 3:**
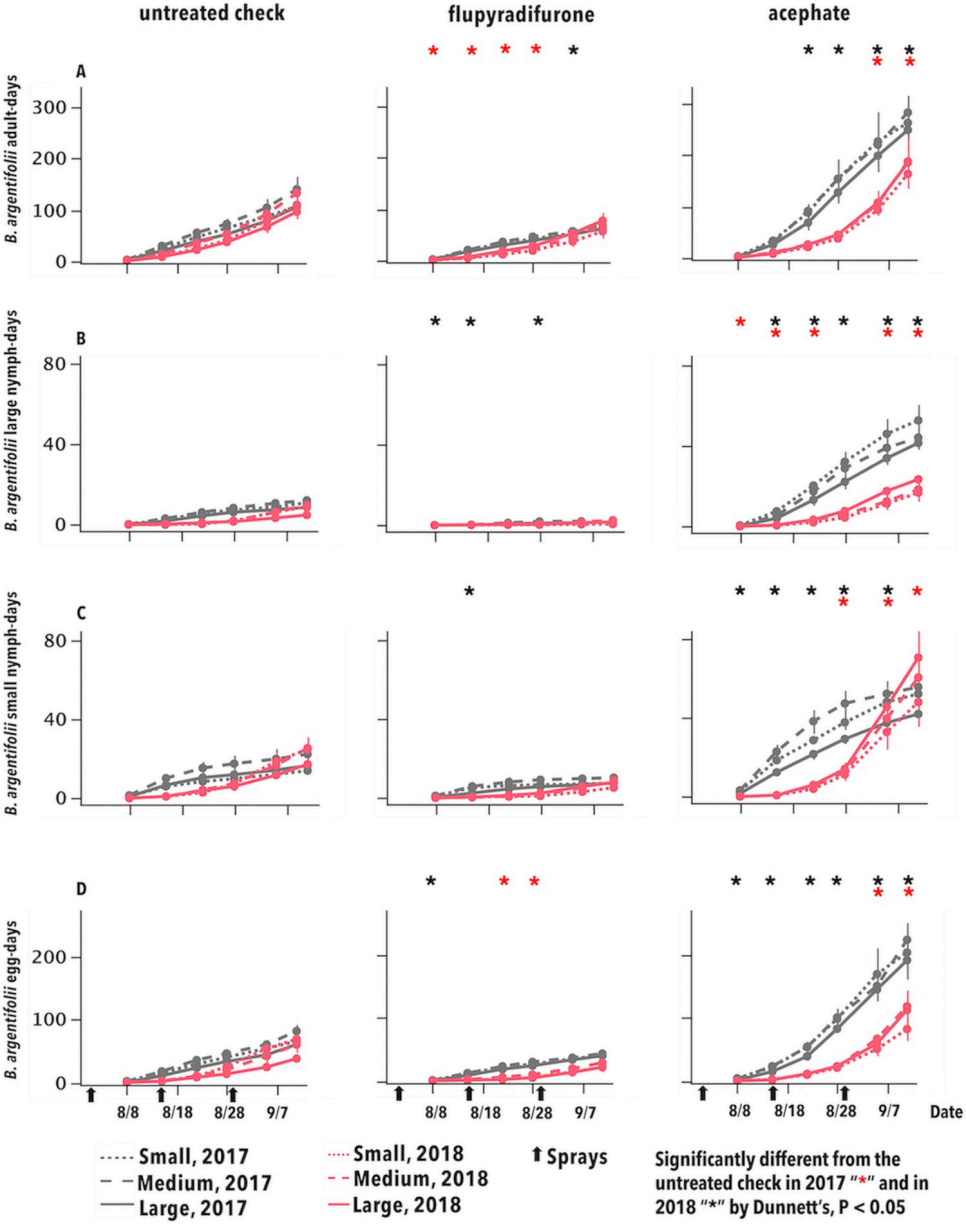
Cumulative mean insect-days (error bars = S.E.) for *B*. *argentifolii*, expressed as number of adults per leaf (A), large (B), small (C) nymphs and (D) eggs per 3.88 cm^2^ leaf disc. Asterisks correspond to treatment means for the main effect of insecticides that were significantly different from the untreated check by Dunnett’s, P < 0.05, by date and year.

The abundance of *B*. *argentifolii* was significantly higher in the positive control than the UTC for several instances in both years across all life stages (Fig 3; S6 Fig in S1 File). The abundance of *B*. *argentifolii* was significantly lower in the flupyradifurone treatment than the UTC in as many as four instances in 2018 and three instances in 2017 (Fig 3; S6 Fig in S1 File).

**Fig 4:**
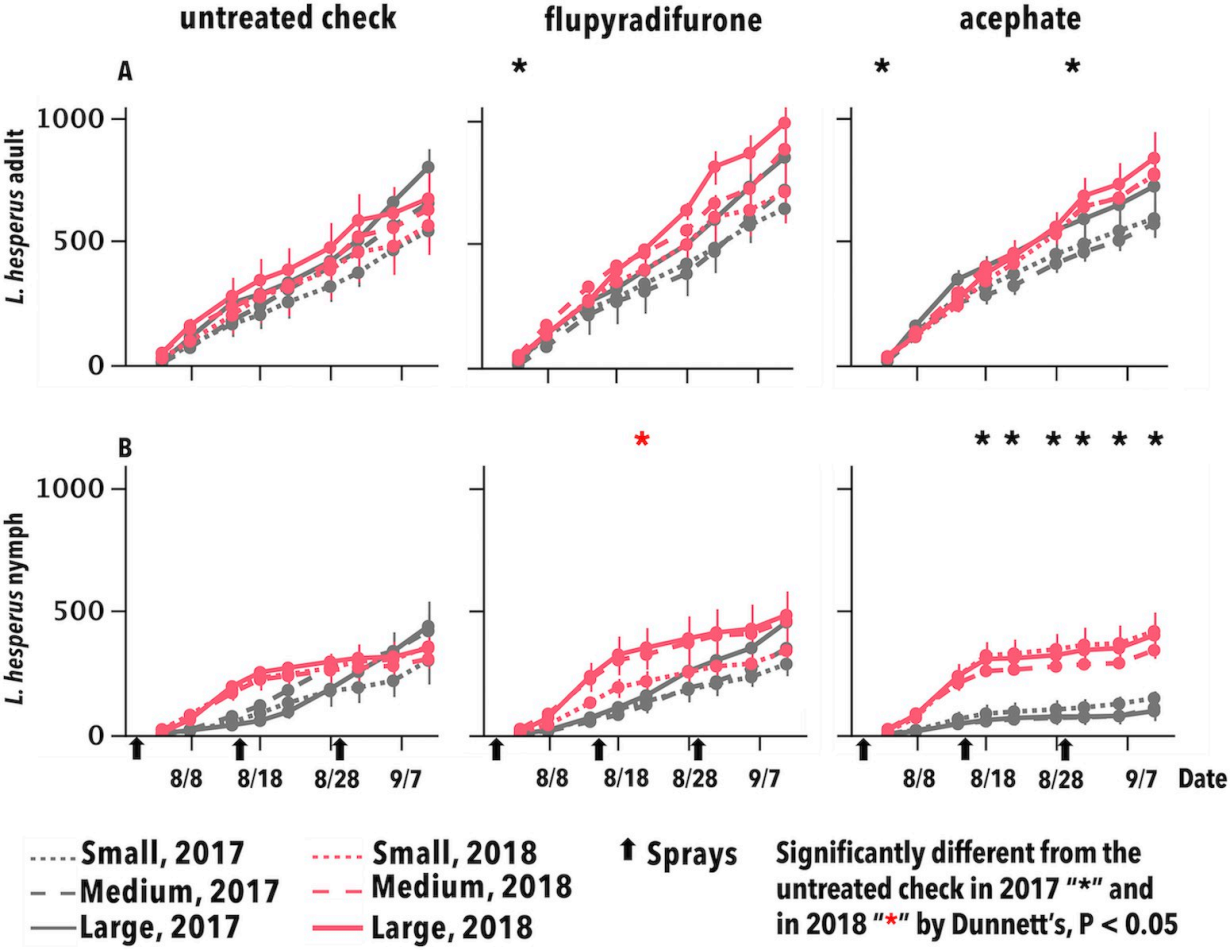
Cumulative mean insect-days for *Lygus hesperus* adults (A) and nymphs (B) per 100 sweeps. Asterisks correspond to treatment means for the main effect of insecticides that were significantly different from the untreated check by Dunnett’s, P < 0.05, by date and year.

Population densities of *L*. *hesperus* were significantly lower in the positive control compared with the UTC on several dates in 2017 (mainly nymphs) and only in one dates in 2018 (Fig 4). Population densities of this pest was significantly higher in the flupyradifurone than the UTC in only one date in both years (Fig 4).

### Biological control function

#### Predator to prey ratios

**Fig 5:**
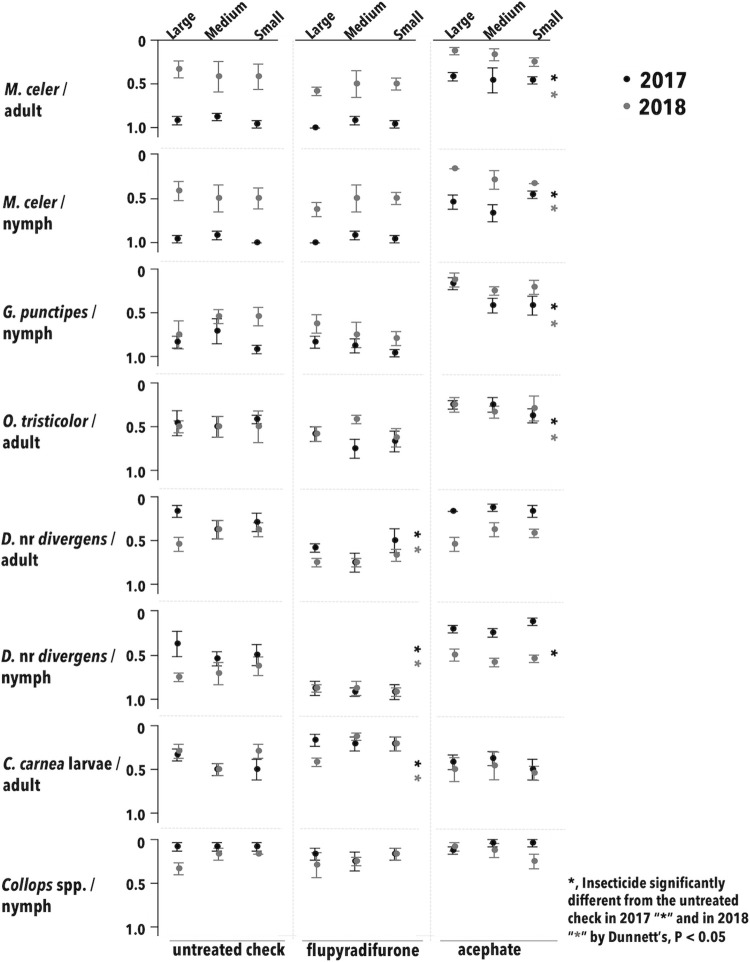
Proportion of time over the season (x-axis) that each of eight predator to prey ratios were above levels indicating functioning biological control (mean ± SE; Vandervoet et al., 2018) for each year. For each insecticide main effect, these proportions were compared with the UTC by Dunnett’s (*, P < 0.05) each year; plot size and its interactions were not significant.

We estimated the proportion of sample dates in which key predator to whitefly ratios were at or above levels indicative of functioning biological control in our system. Plot size and its interactions were not significant (P > 0.05; Table 2). The positive control performed as expected. The proportion of time at or above functioning biological control levels of multiple ratios were significantly lower in the positive control compared to the UTC (Fig 5). This metric in the flupyradifurone treatment was significantly higher or not different from the UTC, with the exception of the ratio *C*. *carnea* larvae to *B*. *argentifolii* adult, in which the proportion of times was significantly lower than the UTC (Fig 5).

**Table 2:** Fixed effect *F*-values of proportion of time that each of the eight predator to prey ratios were above levels associated with biological control of *B*. *argentifolii* nymphs or adults in the Arizona cotton system.

Fixed Factors	DF	*M*. *celer/* nymph	*M*. *celer/* adult	*D*. *nr divergens/* nymph	*D*. *nr divergens/* adult	*G*. *punctipes/* nymph	*O*. *tristicolor/* adult	*C*. *carnea larvae/* adult	*Collops* spp.*/* nymph
Insecticide	2, 54	31.76***	41.83***	69.17***	37.48***	57.15***	14.25***	11.84***	3.49*
Plot Size	2, 54	0.04	0.27	0.68	0.98	1.19	0.25	0.08	0.43
Insecticide*Plot Size	4, 54	1.20	0.49	0.25	1.12	2.01	0.21	1.89	0.89
Year	1, 54	107.71***	101.01***	25.26***	24.49***	14.93***	0.33	0.11	7.37**
Insecticide*Year	2, 54	2.20	3.61*	8.26**	2.13	0.21	1.08	1.33	0.76
Plot Size*Year	2, 54	0.28	0.09	0.30	3.00	0.86	0.36	1.11	0.27
Insecticide*Plot Size*Year	4, 54	0.87	0.19	0.77	0.61	0.47	0.82	0.77	1.46

Mixed-model ANOVA

* *P* < 0.05

** *P* < 0.01

*** *P* < 0.001.

#### Sentinel prey

**Fig 6:**
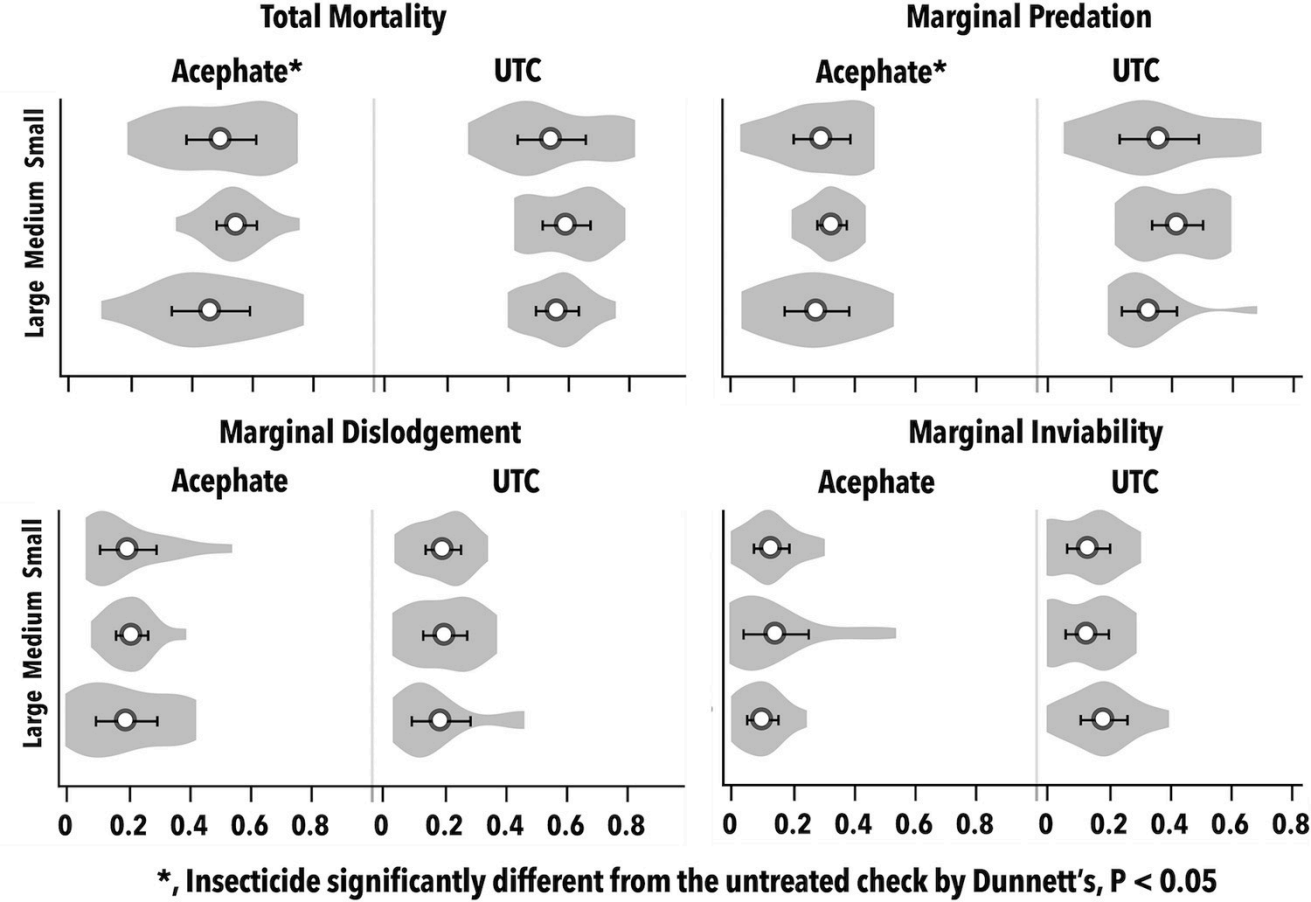
Violin plots showing distributions of mortality rates for *B*. *argentifolii* eggs (mean ± CI). *B*. *argentifolii* are convenient and realistic as sentinel prey, because eggs are immobile on leaves, abundant in the field, and natural enemies readily feed on them. Eggs of *B*. *argentifolii* are one of the most important life stages subjected to biological control in our system. Asterisks denote main effects for insecticides that were significantly different from the untreated check by Dunnett’s, P < 0.05; plot size and its interactions were not significant.

**Fig 7:**
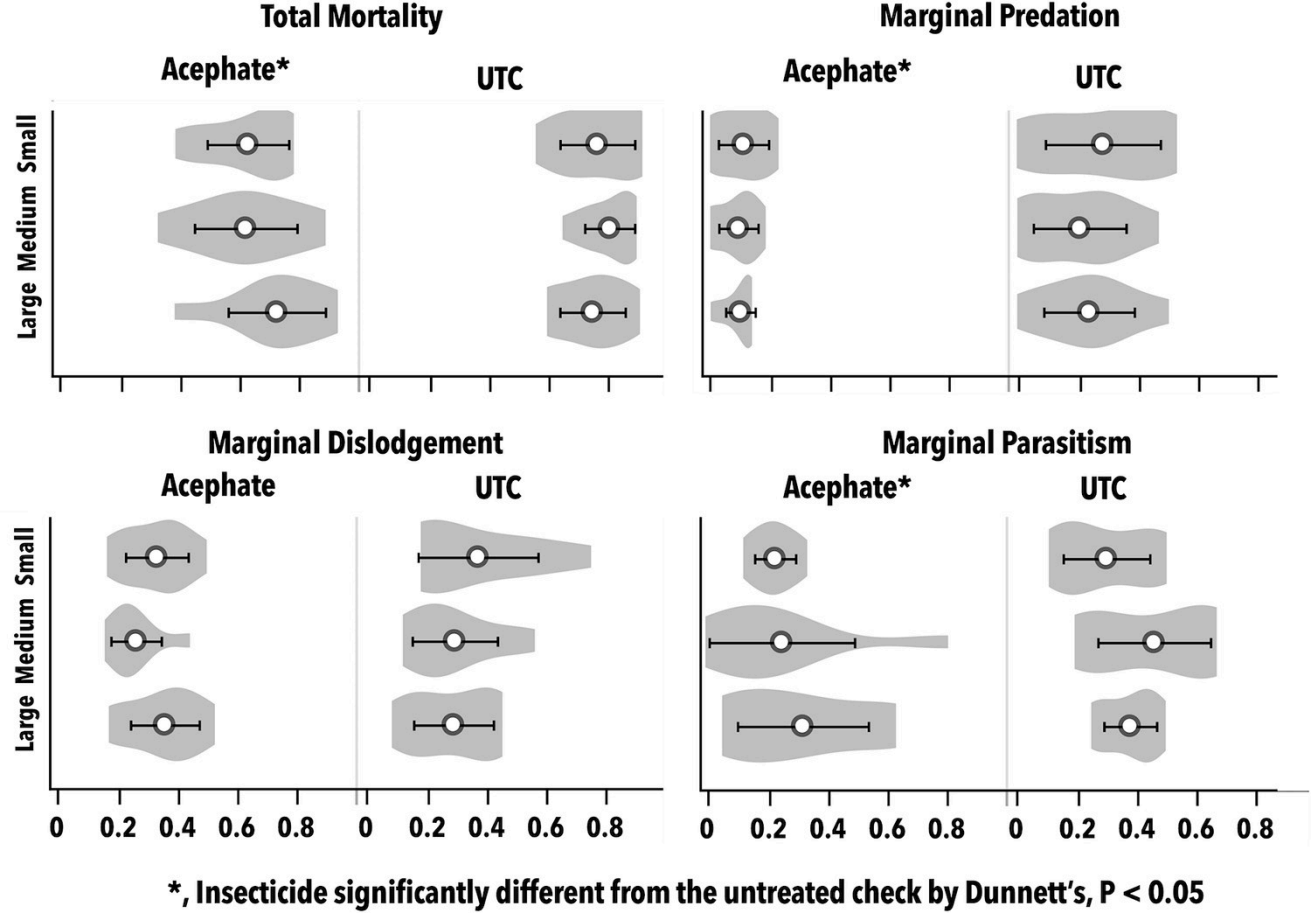
Violin plots showing distributions of rates for each mortality factor for *B*. *argentifolii* nymphs (mean ± CI). *B*. *argentifolii* are convenient and realistic as sentinel prey, because nymphs are immobile on leaves, abundant in the field, and natural enemies readily feed on them. Nymphs of *B*. *argentifolii* are one of the most important life stages subjected to biological control in our system. Asterisks correspond to treatment means for acephate that were significantly different from the untreated check by Dunnett’s, P < 0.05; plot size and its interactions were not significant. Marginal predation in the acephate treatment was significantly different from the untreated check only in the first nymph cohort by Dunnett’s, P < 0.05.

Neither plot size nor any of its interactions were significant for any mortality factor in the sentinel prey study with whitefly eggs or nymphs (P > 0.05; Tables 3 & 4). Total mortality, marginal predation and parasitism (nymphs only) of nymphs and eggs were significantly lower in the positive control than the UTC as expected (Figs 6 & 7).

**Table 3:** Fixed effect *F*-values of mortality factors for *B*. *argentifolii* eggs.

Fixed Factors	DF	Total mortality	Marginal predation	Marginal dislodgement	Marginal inviabiliy
Date	2, 49	9.74**	3.6*	41.52***	6.03**
Insecticide	1, 49	4.61*	4.22*	0.01	0.23
Date*Insecticide	2, 49	2.15	3.01	0.22	1.78
Plot size	2, 49	1.10	1.62	0.17	0.16
Date*Plot size	4, 49	1.80	1.26	1.28	0.23
Insecticide*Plot size	2, 49	0.26	0.09	0.18	0.91
Date*Insecticide*Plot size	4, 49	1.96	1.82	1.14	0.14

Mixed-model ANOVA

* *P* < 0.05

** *P* < 0.01

*** *P* < 0.001.

**Table 4:** Fixed effect *F*-values of mortality factors for *B*. *argentifolii* nymphs.

Fixed Factors	DF	Total mortality	Marginal predation	Marginal dislodgement	Marginal parasitism
Date	1, 30	6.56*	1.43	10.9*	4.34*
Insecticide	1, 28	8.41**	9.13**	0.01	4.5*
Date*Insecticide	1, 28	0.95	4.76*	1.27	0.11
Plot Size	2, 28	0.39	0.33	1.02	0.78
Date*Plot Size	2, 28	0.86	0.30	0.23	0.89
Insecticide*Plot Size	2, 28	2.13	0.14	0.61	0.97
Date*Insecticide*Plot Size	2, 28	3.28	2.53	0.25	1.27

Mixed-model ANOVA

* P < 0.05

** P < 0.01

*** P < 0.001.

#### Diversity indices

**Fig 8:**
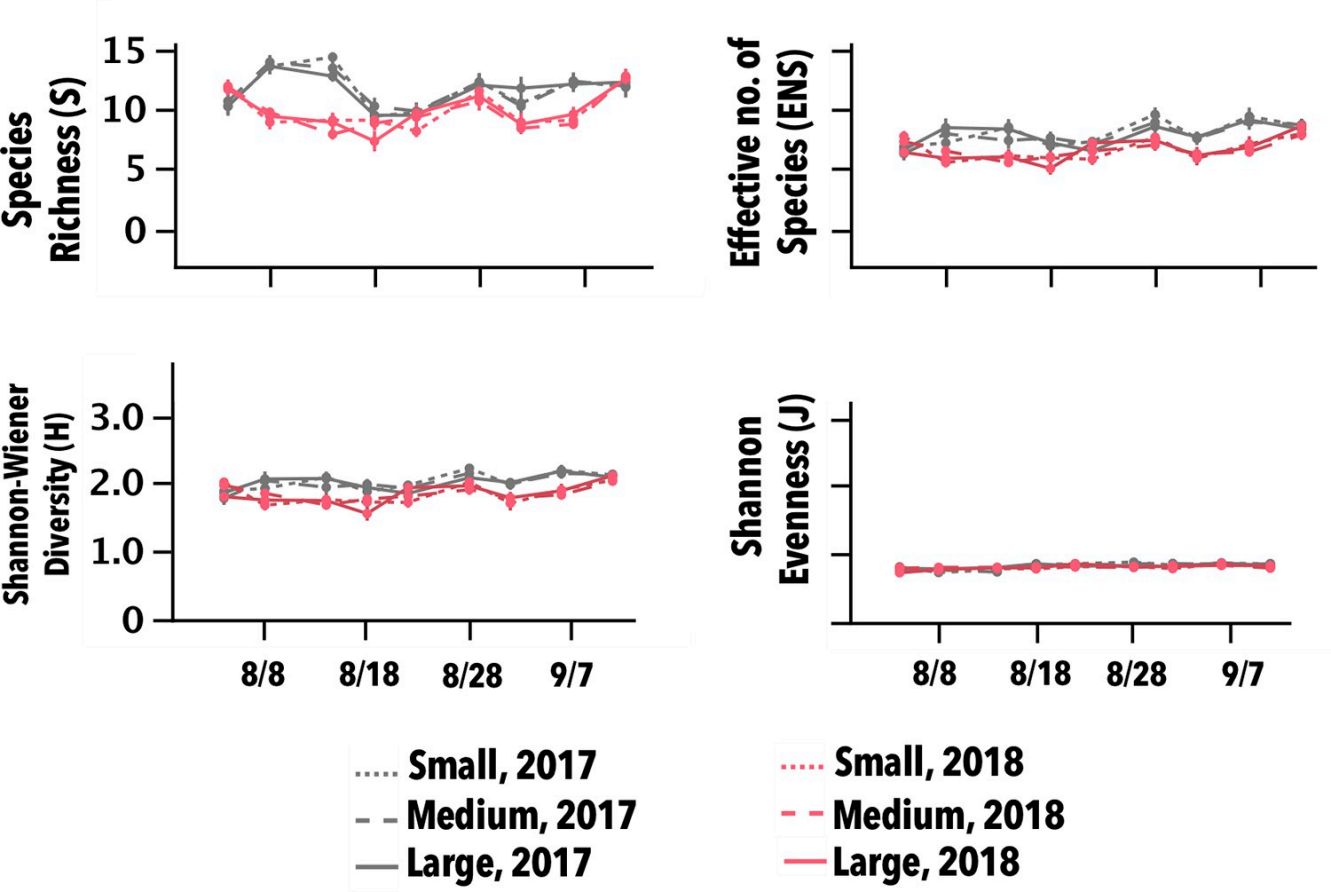
Post-treatment, main effect of plot size over all insecticide treatments (mean ± S.E) for diversity indexes during two growing seasons in Maricopa, AZ. Neither plot size nor any of its interactions were significant (P > 0.05).

None of the diversity indices were significantly affected by plot size or any of its interactions (P > 0.05; S4 Table in S1 File). There also were no strong patterns related to plot size for any of the indices (Fig 8; S5 Table in S1 File).

#### Effect size

Effect size of predator abundance between the positive control acephate or flupyradifurone and the untreated check for each plot size ranged from small (0.2), medium (0.5) to large (0.8) effects sizes using Cohen’s scale [35]. However, there were no patterns related with plot size (Table 5). We also estimated effect size for pest abundance, and there were again no patterns related with plot size (S6 Table in S1 File).

**Table 5:** Effect size (density per 100 sweeps) between acephate or flupyradifurone and the untreated check for each plot size over two growing seasons. Only density is presented for the UTC.

	Large Plot			Medium Plot			Small Plot		
Species	UTC	Acep.	Flupyr.	UTC	Acep.	Flupyr.	UTC	Acep.	Flupyr.
*M*. *celer*	13.56	1.1 (2.89)	0.3 (17.28)	11.9	1 (3.72)	0.3 (10.67)	10.2	1.1 (3.44)	0.6 (10.00)
*G*. *punctipes*	6.00	1.6 (0.39)	0.4 (6.39)	5.28	0.8 (1.39)	0.5 (7.17)	6.56	1.2 (1.44)	0.4 (7.89)
*O*. *tristicolor*	5.72	0.4 (2.94)	0.1 (5.33)	6.28	0.3 (4.61)	0.3 (6.78)	6.50	0.4 (5.78)	0.3 (6.00)
*Drapetis* nr *divergens*	18.11	0.3 (23.33)	0.4 (23.39)	21.5	0.3 (21.28)	0.3 (25.72)	16.9	0.4 (23.11)	0.4 (21.56)
*C*. *carnea* larvae	1.61	0.5 (3.00)	0.3 (1.61)	3.00	0.3 (4.00)	0.7 (1.00)	2.00	0.4 (3.67)	0.6 (1.56)
*Collops* spp.	0.78	0.3 (0.44)	0.4 (0.94)	0.67	0.6 (0.44)	0.4 (1.06)	0.67	0.3 (0.72)	0.3 (0.89)

## Discussion

We investigated how plot size impacts the estimation of treatment effects relative to density, diversity and biological control function for arthropod taxa with a wide range of mobility (Arachnids, Coleoptera, Hemiptera, Diptera, etc.). We found no effect of plot size and concluded that “small” square plots (144 m^2^) are sufficiently large to measure insecticidal effects on non-target arthropods in Arizona cotton. We were able to clearly measure the known destructive effects of the positive control (acephate), and detected effects of the selective insecticide flupyradifurone in small plots, including improved predation and arthropod abundance. Small plots supported arthropods for all parameters compared to medium and large plots based on several metrics: 1) individual species abundance and effect sizes of key predators and pests; 2) community structure (PRCs) and diversity (indices); and 3) biological control function (mortality of sentinel prey) and success (predator to prey ratios).

The use of maintenance sprays in this study were necessary because of the potential differences in prey density due to the three treatment sprays and the different spectrum of control of the selective maintenance insecticide and the positive control [17]. The selective insecticide flupyradifurone has excellent whitefly control, and the positive control acephate is effective against Lygus and destructive to whitefly predators, releasing this pest from biological control. As a result, imbalances in prey level and in plot characteristics (i.e., boll and flower loss due to Lygus and excess honeydew due to whiteflies) could occur and might impact arthropod distribution differently in each insecticidal treatment. However, despite our best efforts to achieve prey parity with maintenance sprays for whiteflies and Lygus, there were still significant differences in prey levels in comparison with the negative control in a few instances. Even with variable abundance of prey among insecticidal treatments that had the potential to impact arthropod distribution in a few cases, results from all tested parameters were consistent across plot sizes, suggesting that our findings are robust to uncontrollable ecological variables.

Previous plot size research done in Texas cotton found that “small” plots were sufficiently large for non-target arthropod studies. Harding et al. [36] studied the effect of plot size on pests and predatory arthropods in cotton plots ranging from ca. 200 to 400 m^2^ treated with aerial sprays, and found that 200 m^2^ plots supported similar abundance of prey and predators compared to larger plots. Their 200 m^2^ plot was only three rows larger than our smallest plot size. However, Harding et al. [36] only sprayed the trial with broad-spectrum insecticides, sampled with a D-vac machine, analyzed arthropods all together, did not report the use of “maintenance sprays” to attempt prey parity among treatments. Further, *L*. *hesperus*, a significant pest in the United States, was not the object of their study at that time. Besides these differences, our study was more robust by also measuring additional important ecological variables such as key predator to whitefly ratios indicating functional biological control in our system along with direct measurements of predation (sentinel prey), and effects on community structure and diversity that included 43 taxa.

Plot size in non-target arthropod studies varies considerably [3]. For example, studies involving non-target species in arable crops used plots ranging from 11,800 to 16,900 m^2^ in corn [37], and some recent studies used plots of 12 m^2^ in soybeans [38], 21 m^2^ in quinoa [39] and 170 and 500 m^2^ in cotton [40, 41]. In meta-analyses to examine the non-target impacts of transgenic Bt crops, Wolfenbarger et al. [42] found no pattern in effect sizes between Bt and non-Bt crops over plot sizes ranging from 20–175,000 m^2^. This finding was recently supported by a comprehensive meta-analysis of Bt maize [43]. In contrast, studies conducted in cereals using a variety of plot sizes suggested that small plot trials may underestimate potential harmful effects of insecticides on non-target arthropods [4–6].

Our small plot here was the smallest practical plot that we could use without compromising sample unit size and over-sampling the fauna in our system. In our small plots, almost the entire length of two middle rows is needed to complete 25 sweeps and still avoid sampling the plot edges. It also was important to have a significant number of interior rows designated for sampling so that one could use alternate rows over time and minimize plant damage caused by sweeps. The minimal plot size is also a factor of the sampling method. Thus, systems that use other sampling methods (i.e., beat sheets) could potentially be subjected to smaller plots whereas other sampling methods might dictate the need for a larger plot. Plot geometry also needs to be considered. Our plots were square, however; long rectangular plots of the same area might not perform well because the distance between field edges of the longest side of rectangular plots would be reduced, which could influence arthropod movement and behavior differently. Distance between plots, plot isolation by alternate crops or bare ground and surrounding vegetation can impact arthropod movement as well [2]. We did not explicitly examine the effects of interplot space, but our results from the small plot size suggest that interplot movement across 3 meters of bare ground at high air temperatures in the summer was inconsequential because we were able to detect treatment differences in these small plots. Ground dwelling arthropods might behave differently, but they were not directly the subject of this investigation.

At the other end of the spectrum, there were practical constraints to testing even larger plot sizes, including significant increases in costs of land rental, irrigation costs, labor, etc. Previous research done in larger plots in Arizona cotton found no effect of plot size in a long-term study testing the effects of transgenic Bt cotton on non-target arthropod abundance between plots ranging from 1200 m^2^ to 20,000 m^2^ [11]. Larger plots are normally more heterogenous and might require greater sampling effort or more replicates to detect changes in population density [11]. Another factor that needs to be considered is the potential loss of homogeneity of blocks as larger plots are used. Blocking field experiments control for nuisance variations like irrigation, soil texture, adjacent fields; however, at some point, inter-plot differences risk being larger than block differences or blocks cannot be efficiently established.

This study investigated the effect of plot size on several taxa with a wide range of mobility in Arizona cotton. We demonstrated that “small” square plots (144 m^2^) are sufficiently large for non-target arthropod studies in Arizona cotton. Small plots allowed us to detect treatment differences, and supported similar individual predator abundance, arthropod community structure and diversity, and biological control function and success compared with larger plot sizes. Therefore, our results are applicable to cotton systems with related pests, predators or other fauna with similar dispersal characteristics. These findings can be applied to target and non-target organism studies in cotton, including studies involving transgenic cotton with insecticidal properties. Moreover, these results also might be generalizable to other crop systems with similar fauna.

This new information should be helpful to growers, researchers, technology providers and regulatory agencies in measuring impacts of various insect control technologies on non-target arthropods in cotton. Furthermore, they point to a scale of testing that should be considered when developing any IPM guidelines that are provided to farmers for use under commercial conditions. These results will guide Arizona’s field evaluation of current and future technologies with goals of providing reliable information about risk to non-target arthropods to our growers [44].

## Supporting information

S1 File Supplemental information contains all the supporting tables and figures. (PDF)
