# Supplementary material for: Spatial scale of non-target effects of cotton insecticides
Source: PLoS One. 2023 May 10;18(5):e0272831. doi: 10.1371/journal.pone.0272831 (PMC10171601; doi:10.1371/journal.pone.0272831)
Supplement: S1 File — (PDF) [file pone.0272831.s001.pdf]

S1 Table. Maintenance sprays for prey parity across insecticidal treatments.

Spray Date	Insecticide	Target	untreated check	flupyradifurone	acephate
8/10/17	flonicamid	*L. hesperus*	•	•	
8/24/17	flonicamid	*L. hesperus*	•	•	
8/02/18	flonicamid	*L. hesperus*	•	•	
8/16/18	flonicamid	*L. hesperus*	•	•	
8/30/18	flonicamid	*L. hesperus*	•	•	
8/24/17	pyriproxyfen	*B. argentifolii*	•		•
8/30/18	buprofezin	*B. argentifolii*	•		•

- Insecticidal treatments that received maintenance sprays.

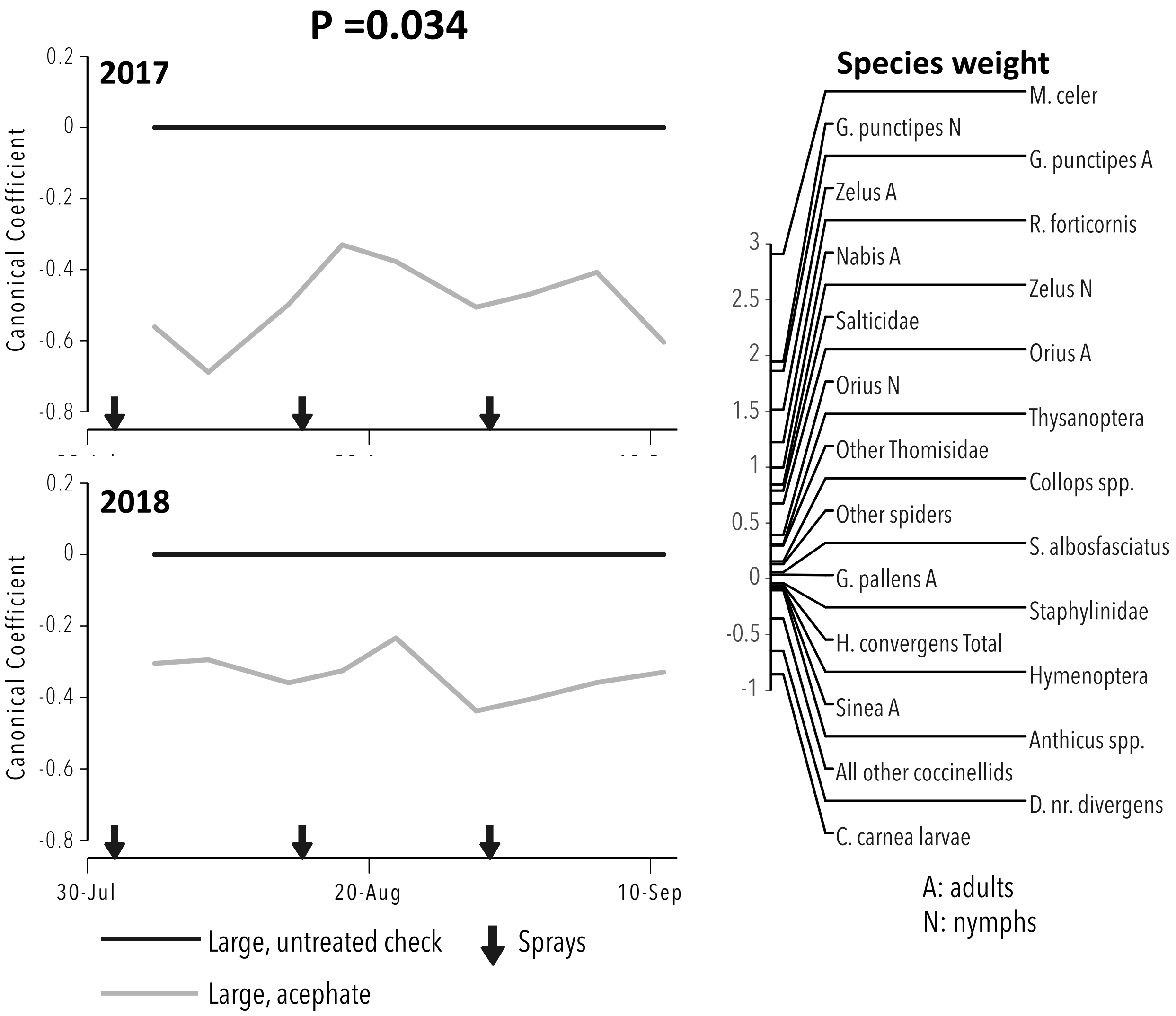

S1 Fig. Principal response curves (PRC) showing the effect of acephate on the arthropod community relative to the untreated check (y = 0 line) for the large plot size during two growing

seasons in Maricopa, AZ. The P-value, P = 0.034, denotes the significance of the PRC analysis over all dates based on an F-type permutation test. The product of the species weight and the canonical coefficient for a given insecticide and time estimates the natural log change in density of that species relative to the untreated check. The greater the species weight the more the response for that species resembles the PRC. Negative weights indicate an opposite pattern, and weights between −0.5 and 0.5 indicate a weak response or a response unrelated to the PRC.

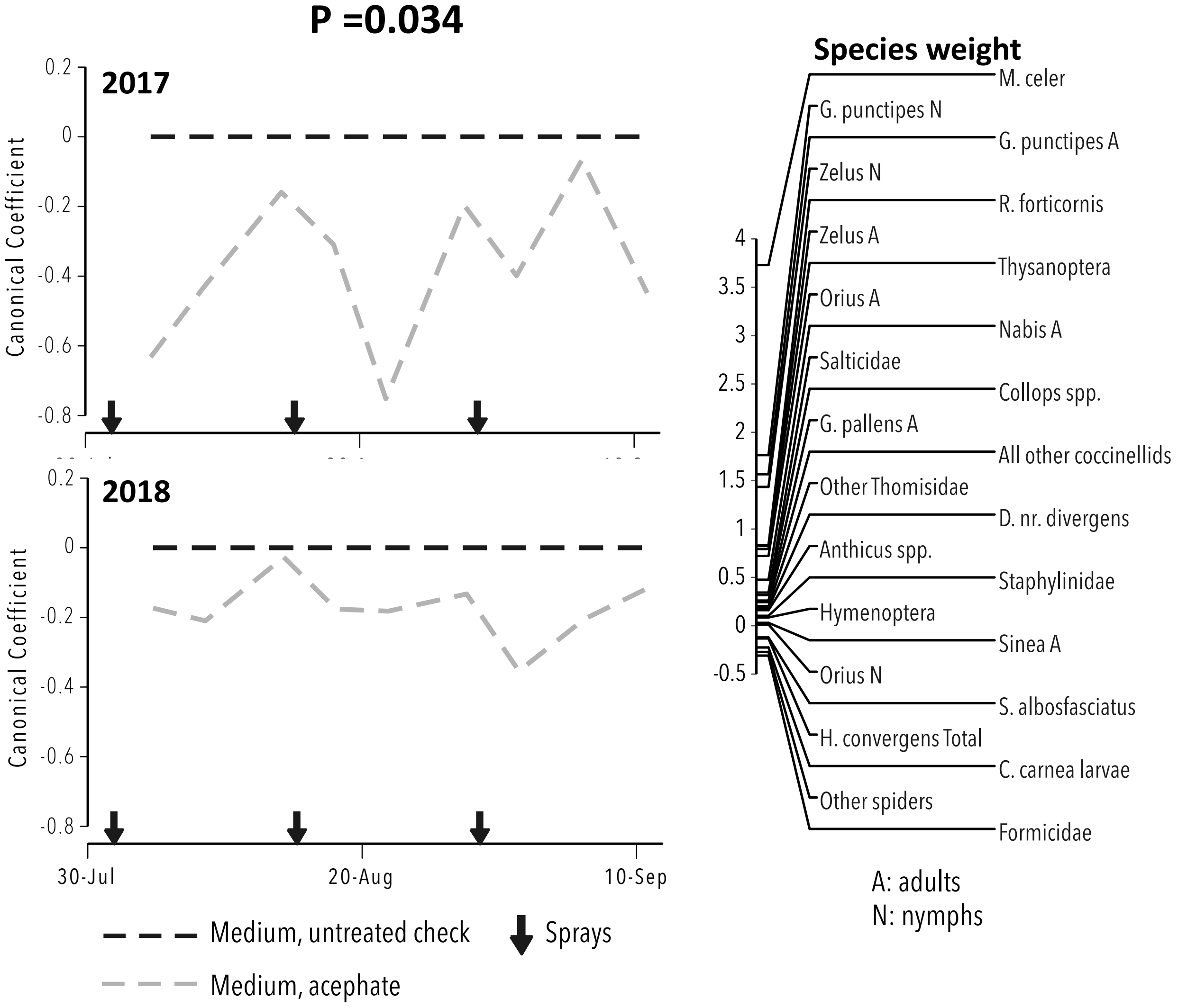

S2 Fig. Principal response curves (PRC) showing the effect of acephate on the arthropod community relative to the untreated check (y = 0 line) for the medium plot size during two growing seasons in Maricopa, AZ. The P-value, P = 0.034, denotes the significance of the PRC analysis over all dates based on an F-type permutation test. The product of the species weight and the canonical coefficient for a given insecticide and time estimates the natural log change in density of that species relative to the untreated check. The greater the species weight the more the response for that species resembles the PRC. Negative weights indicate an opposite pattern, and weights between −0.5 and 0.5 indicate a weak response or a response unrelated to the PRC.

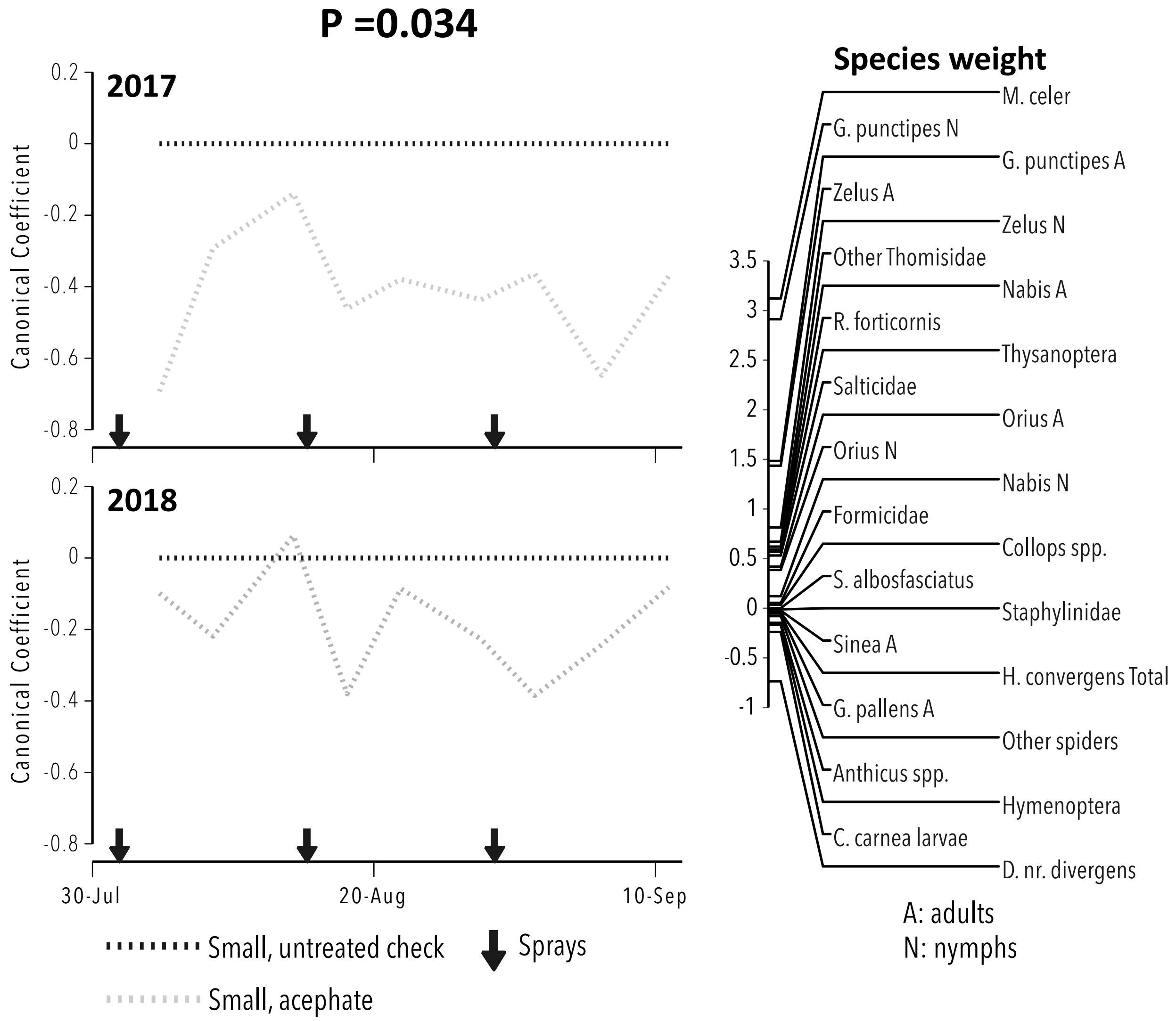

S3 Fig. Principal response curves (PRC) showing the effect of acephate on the arthropod community relative to the untreated check (y = 0 line) for the small plot size during two growing seasons in Maricopa, AZ. The P-value, P = 0.034, denotes the significance of the PRC analysis over all dates based on an F-type permutation test. The product of the species weight and the canonical coefficient for a given insecticide and time estimates the natural log change in density of that species relative to the untreated check. The greater the species weight the more the response for that species resembles the PRC. Negative weights indicate an opposite pattern, and weights between −0.5 and 0.5 indicate a weak response or a response unrelated to the PRC.

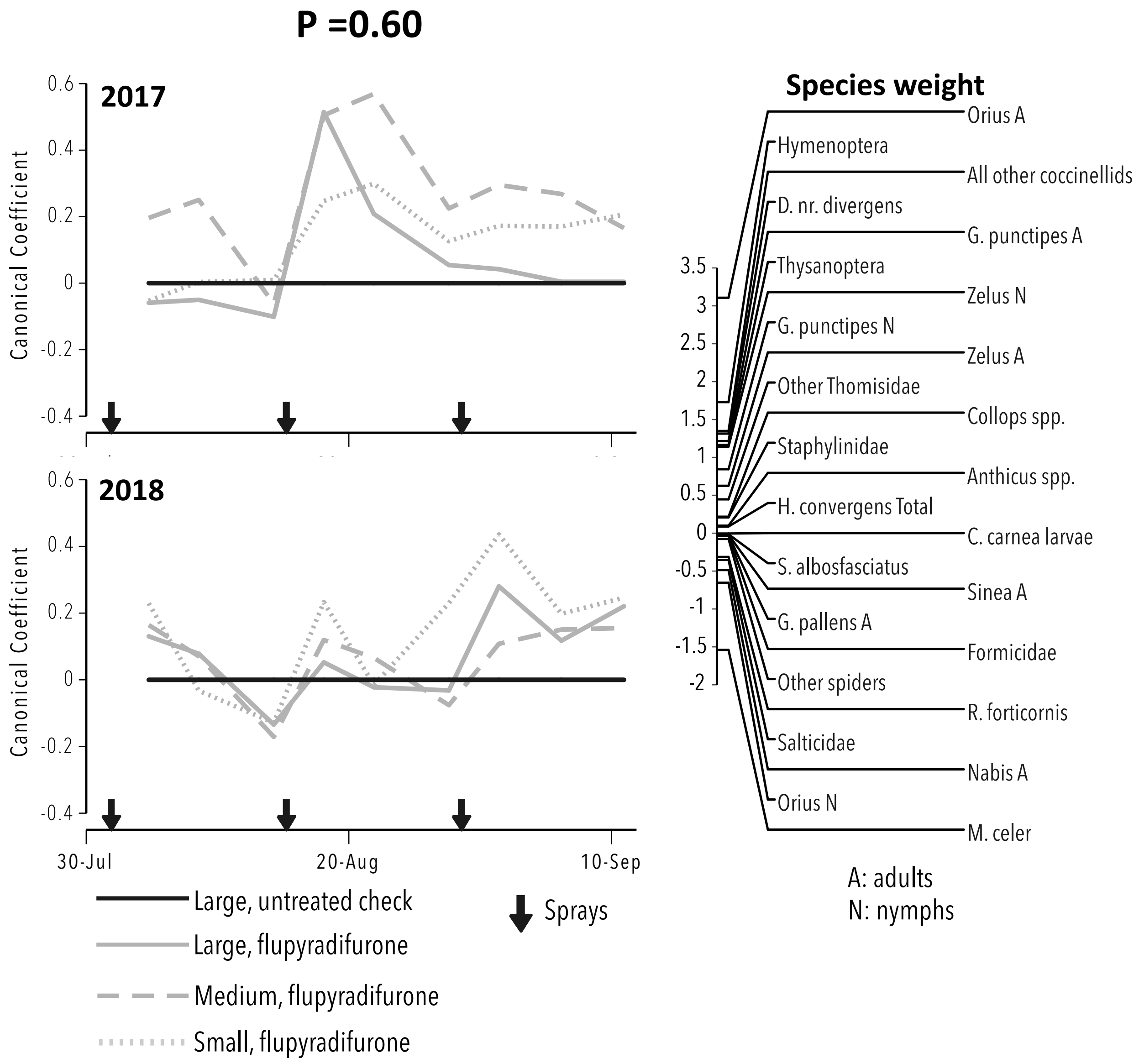

S4 Fig. Principal response curves (PRC) showing the effect of flupyradifurone on the arthropod community relative to the untreated check (y = 0 line) for all plot sizes during two growing seasons in Maricopa, AZ. The P-value, P = 0.60, denotes the significance of the PRC analysis over all dates based on an F-type permutation test. The product of the species weight and the canonical coefficient for a given insecticide and time estimates the natural log change in density of that species relative to the untreated check. The greater the species weight the more the response for that species resembles the PRC. Negative weights indicate an opposite pattern, and weights between −0.5 and 0.5 indicate a weak response or a response unrelated to the PRC.

S2 Table. Fixed effect F-values for mean *Bemisia argentifolii* abundance over two years.

Fixed Factors	DF	Adult	Large nymph	Small nymph	Egg
Insecticide	2, 52	57.03***	190.63***	90.51***	62.83***
Plot Size	2, 52	1.41	0.47	1.30	1.35
Year	1, 52	6.39*	56.05***	0.08	2.20
Date	5, 217.6	23.86***	11.72***	10.08***	24.80***
Insecticide*Plot Size	4, 52	0.26	0.75	0.21	0.81
Insecticide*Date	10, 239.1	6.59***	6.82***	3.38***	13.00***
Insecticide*Year	2, 52	3.90*	38.27***	0.37	7.01**
Insecticide*Year*Date	10, 239.1	4.78***	3.20***	11.71***	1.35
Plot Size*Year	2, 52	0.20	0.95	1.03	0.26
Plot Size*Date	10, 239.1	1.12	0.22	1.87	3.02**
Plot Size*Year*Date	10, 239.1	0.34	0.85	2.01*	3.58***
Date*Year	5, 217.6	6.64***	6.87***	42.26***	4.15**
Insecticide*Plot Size*Date	20, 251.8	0.45	0.40	0.86	1.91*
Insecticide*Plot Size*Year	4, 52.1	0.07	1.44	1.38	0.75

Repeated-measures ANOVA, * $P < 0.05$; ** $P < 0.01$; *** $P < 0.001$. DF are approximated.

B. argentifolii nymphs were classified as small (first and second instars) and large (third and fourth instars, including pharate adults or “pupae”).

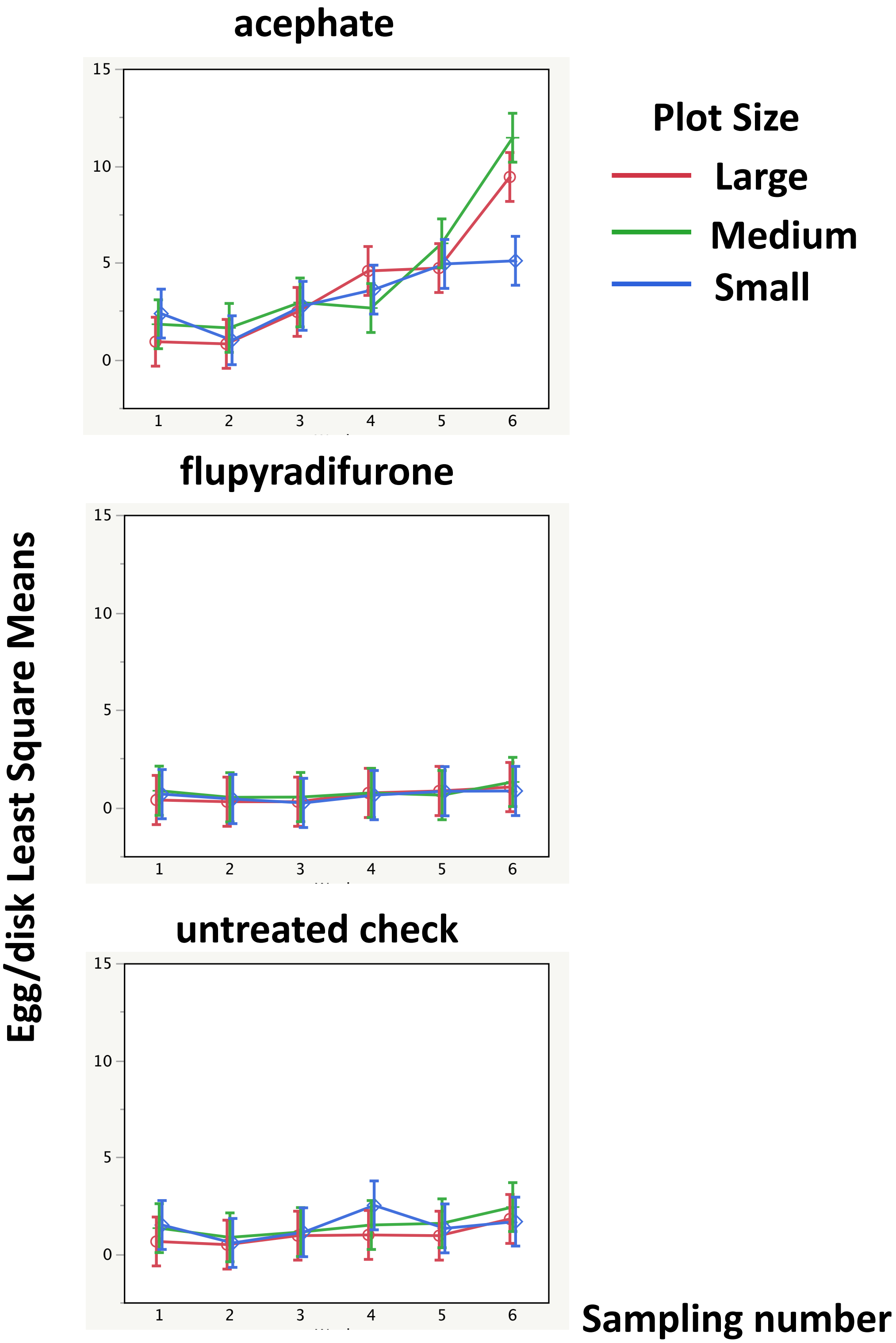

S5 Fig. Least square means of *B. argentifolii* egg per leaf disc.

S3 Table. Fixed effect F-values for mean *Lygus hesperus* abundance (per 100 sweeps) over two years.

**Fixed Factors**	**DF**	**Adult**	**Nymph**
Insecticide	2, 54	4.99*	6.70*
Plot Size	2, 54	6.54*	1.00
Year	1, 54	8.17*	6.61*
Date	8, 464	27.76***	23.4***
Insecticide*Plot Size	4, 54	0.44	0.87
Insecticide*Date	16, 464	1.79*	2.21*
Insecticide*Year	2, 54	1.78	7.82*
Insecticide*Year*Date	16, 464	2.10*	1.15
Plot Size*Year	2, 54	0.43	0.19
Plot Size*Date	16, 464	0.50	1.23
Plot Size*Year*Date	16, 464	1.51	1.47
Date*Year	8, 464	15.28***	21.82***
Insecticide*Plot Size*Date	32, 464	0.70	0.82
Insecticide*Plot Size*Year	4, 54	0.13	0.56

Repeated-measures ANOVA, * $P < 0.05$; ** $P < 0.01$; *** $P < 0.001$. DF are approximated.

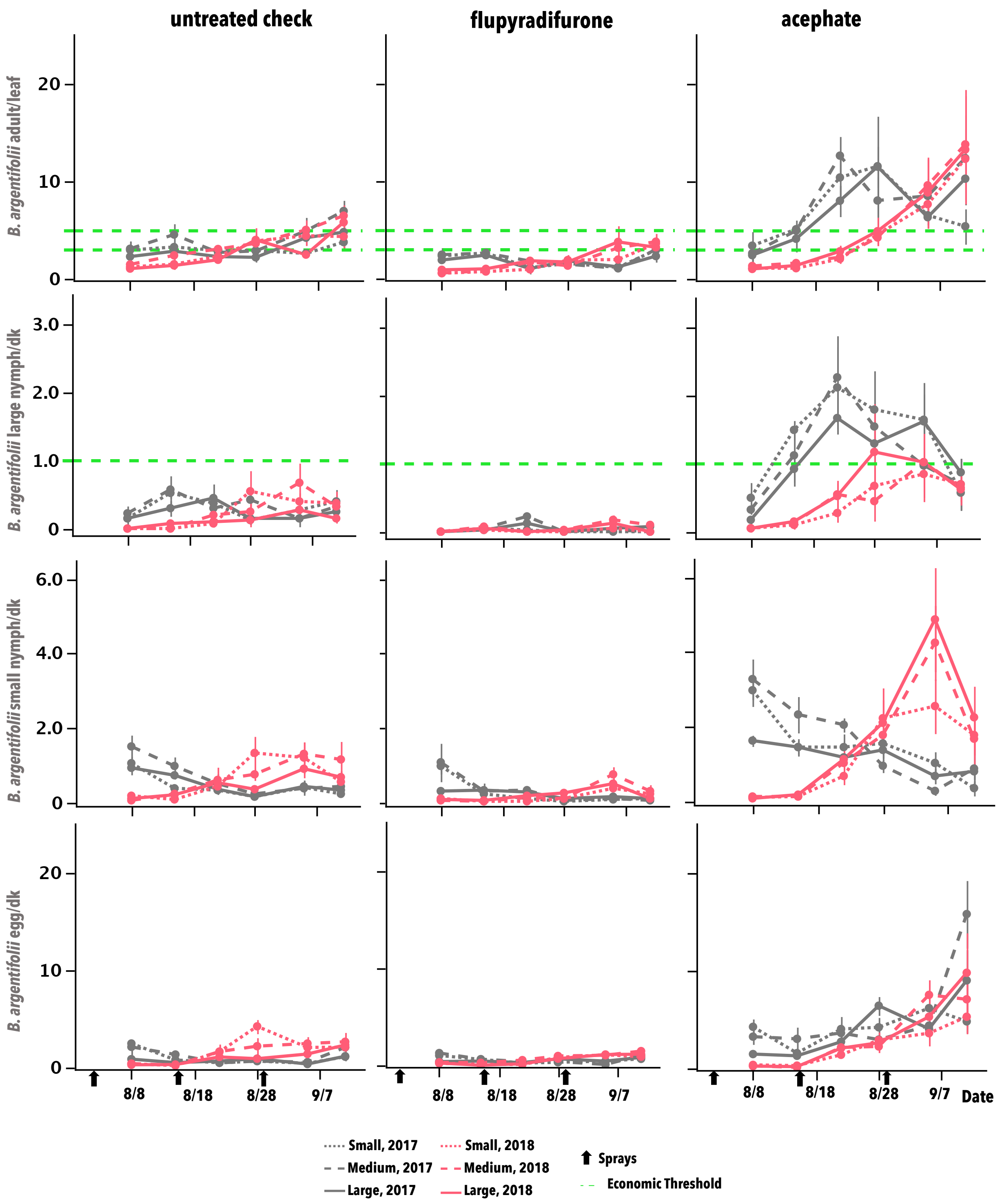

S6 Fig. Post-treatment, weekly mean densities (error bars = S.E.) of *B. argentifolii,* expressed as number of adults per leaf, large, small nymphs and eggs per 3.88 cm^2 leaf disc.

S4 Table. Fixed effect F-values of mean seasonal biodiversity indices.

Fixed Factors	DF	Species Richness (S)	Shannon-Wiener Diversity (H)	Effective no. of Species (ENS)	Shannon Evenness (J)
Insecticide	2, 54	22.25***	9.89***	11.42***	10.1***
Plot Size	2, 54	0.02	0.05	0.15	0.00
Year	1, 54	59.08***	57.13***	61.62***	5.38*
Date	8, 383	22.63***	11.84***	12.59***	10.21***
Insecticide*Plot Size	4, 54	1.34	0.75	0.60	0.28
Insecticide*Date	16, 410	2.75***	2.13***	1.75*	1.28
Insecticide*Year	2, 54	2.38	0.58	1.09	0.39
Insecticide*Year*Date	16, 410	0.92	1.44	1.52	1.68*
Plot Size*Year	2, 54	0.10	0.11	0.06	0.46
Plot Size*Date	16, 410	0.91	1.34	1.16	1.25
Plot Size*Year*Date	16, 410	0.73	0.96	0.78	1.19
Date*Year	8, 383	20.98***	5.48***	6.2***	2.54*
Insecticide*Plot Size*Date	32, 421	1.06	1.04	1.13	0.94
Insecticide*Plot Size*Year	4, 54	1.17	1.26	1.20	0.38

Repeated-measures ANOVA, * $P < 0.05$; ** $P < 0.01$; *** $P < 0.001$. DF are approximated.

S5 Table. Seasonal mean biodiversity indices for each treatment over two years (means ± SE).

Insecticide	Plot Size	Species Richness (S)	Effective no. of Species (ENS)	Shannon-Wiener Diversity (H)	Shannon Evenness (J)
acephate	Large	9.2 ± 0.6	6.6 ± 0.6	1.8 ± 0.1	0.8 ± <0.1
acephate	Medium	9.9 ± 0.4	7.0 ± 0.4	1.9 ± <0.1	0.8 ± <0.1
acephate	Small	10.1 ± 0.5	7.1 ± 0.4	1.9 ± <0.1	0.8 ± <0.1
flupyradifurone	Large	11.3 ± 0.8	7.2 ± 0.5	1.9 ± <0.1	0.8 ± <0.1
flupyradifurone	Medium	11.3 ± 1.4	7.0 ± 0.7	1.9 ± 0.1	0.8 ± <0.01
flupyradifurone	Small	11.2 ± 0.6	7.3 ± 0.4	1.9 ± <0.1	0.8 ± <0.1
UTC	Large	11.7 ± 0.9	8.0 ± 0.6	2.0 ± <0.1	0.8 ± <0.01
UTC	Medium	11.1 ± 0.9	7.7 ± 0.6	2.0 ± <0.1	0.8 ± <0.01
UTC	Small	11.1 ± 1.3	7.8 ± 1.1	2.0 ± 0.1	0.8 ± <0.1

S6 Table. Effect size (density per 100 sweeps) between acephate or flupyradifurone and the untreated check for each plot size over two growing seasons. Only density is presented for the UTC.

	Large Plot			Medium Plot			Small Plot		
**Species life stage**	**UTC**	**Acep.**	**Flupyr.**	**UTC**	**Acep.**	**Flupyr.**	**UTC**	**Acep.**	**Flupyr.**
B. argentifolii adult	2.98	0.8 (6.27)	0.6 (1.97)	3.96	0.7 (6.85)	1.4 (2.02)	3.05	0.8 (5.95)	0.9 (1.83)
B. argentifolii large nymph	0.18	1.4 (0.84)	1.1 (0.04)	0.29	0.8 (0.82)	1.1 (0.06)	0.27	1.0 (0.90)	1.4 (0.01)
B. argentifolii small nymph	0.47	1.3 (1.48)	1.1 (0.20)	0.67	0.8 (1.56)	0.8 (0.27)	0.52	1.0 (1.35)	0.8 (0.20)
B. argentifolii egg	0.96	1.1 (3.81)	0.6 (0.59)	1.46	1.0 (4.41)	0.7 (0.76)	1.44	1.2 (3.29)	0.8 (0.60)
L. hesperus adult	19.28	0.4 (19.89)	0.4 (23.94)	16.67	0.4 (17.22)	0.5 (20.94)	14.22	0.4 (17.22)	0.5 (17.50)
L. hesperus large nymph	4.22	0.7 (2.11)	0.5 (4.94)	3.67	0.5 (2.17)	0.4 (4.56)	2.61	0.6 (2.33)	0.4 (3.61)
L. hesperus small nymph	5.89	0.6 (4.28)	0.2 (7.00)	5.83	0.5 (4.00)	0.4 (5.94)	6.39	0.4 (4.67)	0.4 (4.33)

B. argentifolii nymphs were classified as small (first and second instars) and large (third and fourth instars, including pharate adults or "pupae"). *L. hesperus* nymphs were considered as either small (first and second instars) or large (third to fifth instars).
